# An Energy Harvester Coupled with a Triboelectric Mechanism and Electrostatic Mechanism for Biomechanical Energy Harvesting

**DOI:** 10.3390/nano12060933

**Published:** 2022-03-11

**Authors:** Lei Zhai, Lingxiao Gao, Ziying Wang, Kejie Dai, Shuai Wu, Xiaojing Mu

**Affiliations:** 1 School of Mechanical Engineering, Hebei University of Technology, Tianjin 300401, China; zlhbgd@163.com (L.Z.); wushuai@hebut.edu.cn (S.W.); 2 School of Electrical and Mechanical Engineering, Pingdingshan University, Pingdingshan 467000, China; dkj671@163.com; 3 Key Laboratory of Optoelectronic Technology & Systems, Ministry of Education, International R&D Center of Micro-Nano Systems and New Materials Technology, Chongqing University, Chongqing 400044, China

**Keywords:** energy harvesting, triboelectric mechanism, electrostatic mechanism, human motion status monitoring

## Abstract

Energy-harvesting devices based on a single energy conversion mechanism generally have a low output and low conversion efficiency. To solve this problem, an energy harvester coupled with a triboelectric mechanism and electrostatic mechanism for biomechanical energy harvesting is presented. The output performances of the device coupled with a triboelectric mechanism and electrostatic mechanism were systematically studied through principle analysis, simulation, and experimental demonstration. Experiments showed that the output performance of the device was greatly improved by coupling the electrostatic induction mechanisms, and a sustainable and enhanced peak power of approximately 289 μW was produced when the external impedance was 100 MΩ, which gave over a 46-fold enhancement to the conventional single triboelectric conversion mechanism. Moreover, it showed higher resolution for motion states compared with the conventional triboelectric nanogenerator, and can precisely and constantly monitor and distinguish various motion states, including stepping, walking, running, and jumping. Furthermore, it can charge a capacitor of 10 μF to 3 V within 2 min and light up 16 LEDs. On this basis, a self-powered access control system, based on gait recognition, was successfully demonstrated. This work proposes a novel and cost-effective method for biomechanical energy harvesting, which provides a more convenient choice for human motion status monitoring and can be widely used in personnel identification systems.

## 1. Introduction

To realize the intellectualization and miniaturization of wearable electronics, the requirements of their power devices have been raised, such as long life, small volume, high energy density, and easy integration [1,2,3,4,5]. Traditional chemical batteries cannot satisfy the requirements of the fast development of wearable electronics due to their fatal defects, such as their large size, non-flexible structure, limited lifetime, and potential hazards to environments [6]. Biomechanical energy is generated in our daily life all the time, including arm swinging, walking, running, and heart beating [7,8,9,10]. If this energy is converted into electricity, it can meet the power consumption for part of wearable electronics [11,12,13,14,15,16,17]. As an emerging energy conversion technology, triboelectric nanogenerators (TENG) have been widely used in the field of self-powered wearable electronics because of their low cost, environmental friendliness, and flexibility [18,19,20]. A TENG can provide an effective approach to converting such biomechanical energy into electrical energy to provide power for part of wearable electronics, thus constructing a self-powered human motion signal monitoring and recognition system [21,22,23].

However, there are two main problems for current devices to harvest biomechanical energy: (1) Most of the devices are based on a single conversion mechanism, leading to low energy conversion efficiency [24,25,26,27,28,29]; and (2) the energy is usually harvested from a single source. The movements of the human body are coordinated by many parts. For example, leg movements are accompanied with arm movements when walking. If the device is integrated onto the human leg, it is difficult to harvest the energy generated by arm movements. Therefore, in order to improve the efficiency of biomechanical energy harvesting, a variety of energy harvesting strategies should be combined to achieve efficiently cooperative work.

To solve this problem, we propose an energy harvester coupled with a triboelectric mechanism and electrostatic mechanism for biomechanical energy harvesting. By exposing the electrodes of the bracelet which is integrated onto the arm, the energy of the swinging arm and the energy generated by the separation and contact between the foot and the ground during movement can be obtained simultaneously. Traditional walking energy acquisition requires the integration of the insole with the power generation function, which has a short lifetime and will induce a certain level of discomfort during use. The scheme proposed in this paper does not need special processing of the sole and can realize the efficient acquisition of triboelectric energy between the sole and the ground when walking, which has the advantages of comfort, low cost, and long lifetime. Moreover, the device, when coupled with a triboelectric mechanism and electrostatic mechanism, showed higher resolution in the sensing of motion states, which provided a more convenient choice for human motion status monitoring and can be widely used in personnel identification systems.

## 2. Results and Discussion

### 2.1. Working Principle

**Figure 1:**
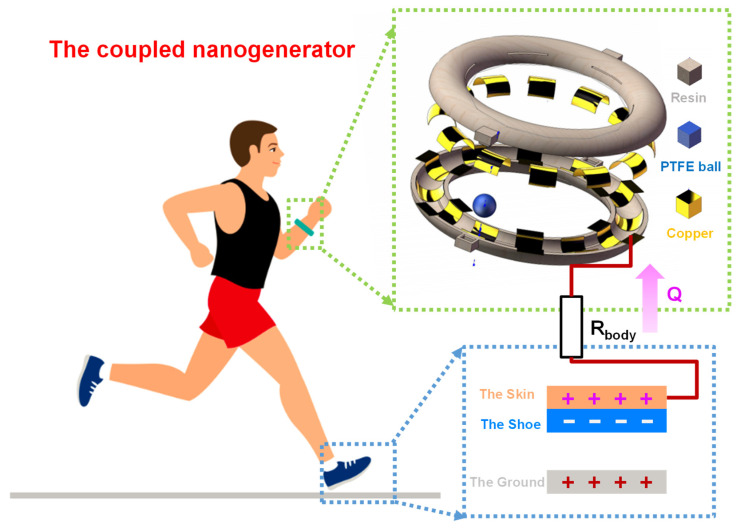
Schematic diagram of the energy harvester coupled with the triboelectric mechanism and electrostatic mechanism for biomechanical energy harvesting.

An energy harvester coupled with a triboelectric mechanism and electrostatic mechanism for biomechanical energy harvesting, as illustrated in Figure 1, is proposed in this paper, which consists of two main parts: a wristband-type triboelectric nanogenerator (W–TENG) worn on the wrist, and an electrostatic nanogenerator (EENG) formed by the friction of the sole against the ground. The structure of the W–TENG, shown in the illustration, mainly consists of three parts: a 3D-printed resin ring, some cross-fingered copper foil electrodes, and a polytetrafluoroethylene (PTFE) ball. Fluorine has the highest electronegativity among almost all the elements, so the material of the ball was PTFE, which contains lots of fluorine [30]. Due to its strong electronic attraction, a PTFE ball can easily capture a large amount of triboelectric charges when rubbed against other materials. Meanwhile, is copper at a more positive direction than precious gold and silver in the triboelectric sequence [31]. Therefore, a PTFE ball and copper electrode will accumulate opposite charges after contact [32]. The whole device can be realized with a simple process and cheap materials which are suitable for mass production. One end of the forked-finger electrode was placed in contact with the human wrist. When the human body moved, the PTFE ball would roll on the cross-fingered electrodes in the 3D-printed resin ring with the swing of the arm, resulting in a triboelectric effect with the cross-fingered electrodes. The EENG was built by the shoe and the floor. In this structure, the sole of the shoe was not installed with any triboelectric materials. The sole and the ground produced a periodic contact—separation process with walking, and opposite charges were accumulated on each other’s surfaces. The surface of the skin can generate electric charges by electrostatic induction because of the contact with the electric shoe. By exposing one end of the electrode of the W–TENG to the human body, the triboelectric charges on the skin are transmitted to the electrode of the W–TENG. The scheme proposed in this paper does not need to place any material in the sole, and can realize the efficient acquisition of triboelectric energy between the sole and the ground when walking, which has the advantages of comfort, low cost, and long life.

**Figure 2:**
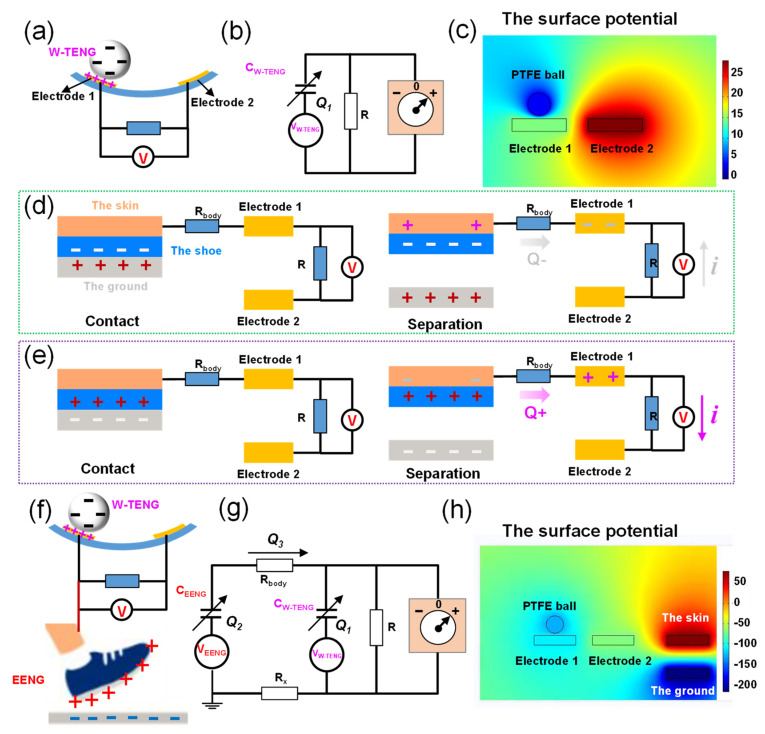
Analysis of the working principle: (**a**) the working principle of the W–TENG; (**b**) the circuit equivalent model of the W–TENG; (**c**) the simulation result of the output of W–TENG; (**d**,**e**) the analysis of charge-transfer direction for different materials; (**f**) the working principle of the C–ENG; (**g**) the circuit equivalent model of the C–ENG; and (**h**) the simulation result of the output of C–ENG.

For the traditional triboelectric nanogenerator (W–TENG), its electrodes were generally shielded inside the shell, as shown in Figure 2a,b. The amount of charges accumulated on the equivalent capacitance *C_W–TENG_* was *Q*_1_ after the PTFE ball contacted with the electrodes, and the open-circuit voltage can be expressed as [33]:

(1) $$V_{OC}=R\frac{dQ_{1}}{dt}+\frac{Q_{1}}{C_{W-TENG}}$$

Due to the motion process of the human body, the sole produces a “contact—separation” process with the ground all the time, and the mechanical energy is converted to electric energy along with this process. The means of capturing this energy effectively is very important. In this work, a mechanism of triboelectric and electrostatic double-effect coupling, to achieve efficient collection of this energy, was proposed. By touching one end of the W–TENG electrode to the human body, the electric charges of the EENG was transmitted to the electrode of the W–TENG through the human body, and the coupled nanogenerator (C–ENG) was built. Indeed, the polarity of the triboelectric charge depends on the polarity of the triboelectric material. Therefore, the charge-transfer processes in two cases were analyzed respectively. When the material of the shoe had more electronegativity than the ground, the shoe accumulated negative charges when it made contact with the ground. Meanwhile, the ground accumulated positive charges, as shown in Figure 2d. When the foot was lifted, the surface of the skin of the human body generated positive charges through electrostatic induction. Due to the fact that the human skin was connected to one electrode (such as electrode one in Figure 2d) of the W–TENG, some of the negative charges were repelled all the way to electrode one. It created a potential difference between electrode one and electrode two of the W–TENG, resulting in a current from electrode two to electrode one in the external circuit. The other case was that the ground had more electronegativity than the material of the shoe, as shown in Figure 2e. As the shoe accumulated positive charges during its contact with the ground, the surface of the skin of the human body generated negative charges through electrostatic induction and some of the positive charges were repelled all the way to electrode one, resulting in a current from electrode one to electrode two in the external circuit. By comparing the two cases, only the direction of the current in the outer circuit changed with the change of the polarity of the material. It should be noted that the soles are usually made of chemical-resistant viton or polyvinyl chloride (PVC), and the floors are usually made of wood (marine-grade plywood) or wear-resistant garolite. Therefore, the soles of the shoes are usually more electronegative than the floors [32].

The working principle of the C–ENG is shown in Figure 2f, and its circuit equivalent model is shown in Figure 2g. As the sole rubbed against the ground, the amount of charge accumulated on the equivalent capacitance (*C_EENG_*) of the EENG was *Q*_2_. Since the amount of triboelectric charge was positively correlated with the friction area, *Q*_2_ was much larger than *Q*_1_ because the sole had a larger area. When the electrode made contact with the human body, part of the charge on *C_EENG_* flowed to *C_W–TENG_*. Assuming that the transferred charge was *Q*_3_, the open-circuit voltage of the device was:

(2) $$V_{OC}=R\frac{d(Q_{1}+Q_{3})}{dt}+\frac{Q_{1}+Q_{3}}{C_{W-TENG}}$$

Therefore, the output performance of the device is improved.

COMSOL 5.4 was employed in the simulation analysis, and the results are illustrated in Figure 2c,h. Figure 2c is the simulation result of the output of the W–TENG. In this figure, the circle represents the PTFE ball that accumulated negative charges, and the rectangles represent the adjacent electrodes that accumulated positive charges. Take the situation in Figure 2d as an example; the simulation result of the output of the C–ENG is shown in Figure 2h. Compared with Figure 2c, Figure 2h added two rectangles at the far right. The top rectangle represents the skin, which accumulated positive charges through electrostatic induction. The bottom rectangle represents the ground, accumulating negative charges.

### 2.2. Electrical Characterization

**Figure 3:**
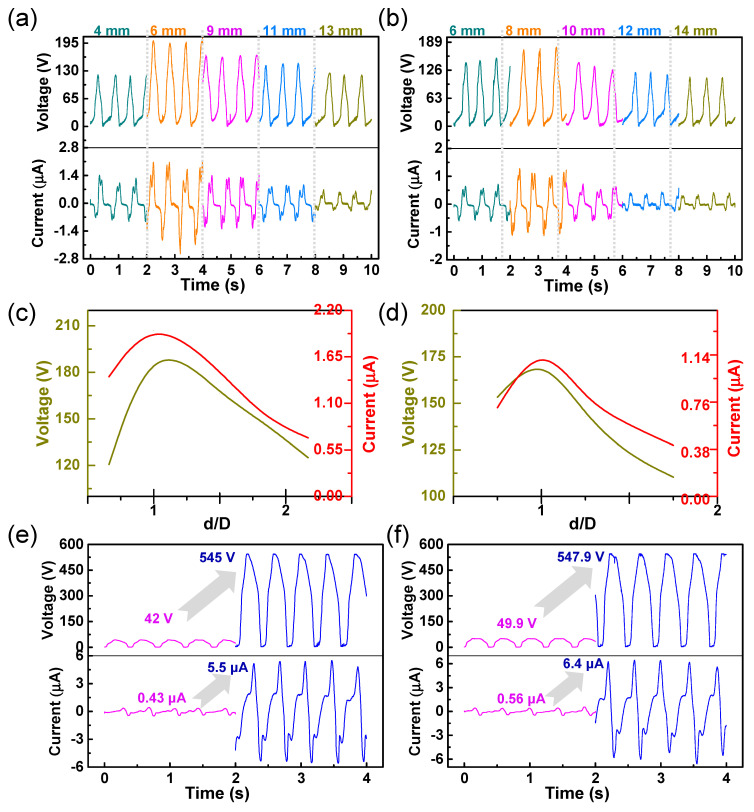
The effects of the coupling electrostatic energy and the relationship between PTFE ball diameter and electrode width on the output of the C–ENG: (**a**) the effect of cross-finger electrode width on the output of the C–ENG at the PTFE ball diameter of 6 mm; (**b**) the effect of cross-finger electrode width on the output of the C–ENG at the PTFE ball diameter of 8 mm; (**c**) a curve fitted according to Figure 3a; (**d**) a curve fitted according to Figure 3b; (**e**) a comparison of output characteristics between W–TENG and C–ENG at the PTFE ball diameter of 6 mm; and (**f**) a comparison of output characteristics between W–TENG and C–ENG at the PTFE ball diameter of 8 mm.

Firstly, in order to analyze the influence of the relationship between the diameter of the PTFE ball and the width of the electrode on the electrical performance of the C–ENG in the state of a human walking with swinging arms, PTFE balls with diameters of 6 and 8 mm were selected as test objects. The output voltages and currents of different electrode widths (4 mm, 6 mm, 9 mm, 11 mm, and 13 mm), with a PTFE ball with a diameter of 6 mm, were measured respectively, as illustrated in Figure 3a. The output voltages and currents of different electrode widths (6 mm, 8 mm, 10 mm, 12 mm, and 14 mm), with a PTFE ball with a diameter of 8 mm, are illustrated in Figure 3b. Figure 3c further analyzes the maximum voltage and current in Figure 3a. Its abscissa represents the ratio of electrode width (d) to PTFE ball diameter (D), and the ordinates on both sides represent the maximum voltage and current under different electrode widths. It can be seen that, when the ratio approached one, both the voltage and current reach their maximum values. The same result is obtained in Figure 3d. These results show that the best electrical properties were obtained when the electrode width was approximately the same as the PTFE ball diameter. After obtaining the above results, the electrical properties in jumping state were analyzed. The voltage and current values were measured when the electrode was not in contact with the human body (W–TENG) and when it was in contact with the human body (C–ENG) respectively. As seen in Figure 3e, when the electrode width was 6 mm and the diameter of the PTFE ball was 6 mm, the output voltages of the W–TENG and C−TENG were 42 V and 545 V respectively. It proves that the output voltage increased by, approximately, 12.98 times by coupling the mechanism of electrostatic. The maximum current correspondingly increased from 0.43 to 5.5 µA. Similarly, it can be seen from Figure 3f that when the electrode width was 8 mm and the diameter of the PTFE ball was 8 mm, the output voltage showed the same increasing trend. The results show that coupling the electrostatic mechanism can effectively improve the energy conversion efficiency of the nanogenerator, which is the same as in the theoretical analysis. In addition, all tests were conducted on the dry ground. This is because, in a dry environment, the sole and the ground can generate more triboelectric charges, which was conducive to verifying the coupling enhancement theory proposed in this paper.

**Figure 4:**
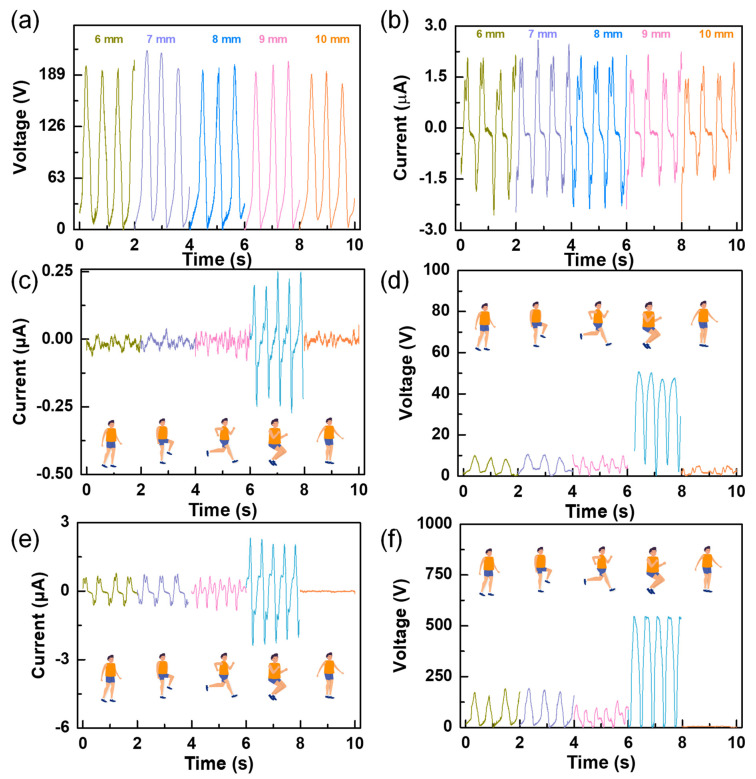
The influence of the diameter of the PTFE ball on the outputs and the outputs of the device under different motion modes: (**a**) the influence of the diameter of the PTFE ball on the output voltage; (**b**) the influence of the diameter of the PTFE ball on the output current; (**c**) the output voltages of W–TENG under different motion modes; (**d**) the output currents of W–TENG under different motion modes; (**e**) the output voltage of C–ENG under different motion modes; and (**f**) the output currents of C–ENG under different motion modes.

In order to further study the influence of the diameter of the PTFE ball on the outputs, other sizes (7 mm, 9 mm, and 10 mm) were measured, under the condition that the size of the PTFE was equal to the width of the electrode, and the measured results were compared with those obtained previously at 6 mm and 8 mm, as illustrated in Figure 4a,b. As seen in the two figures, the diameter of the PTFE ball had no obvious effect on the outputs. This may be because, although the diameter of the ball changed, the total triboelectric area did not significantly change. Through the analysis of the measured experimental data, it can be concluded that the size combination of 7 mm is more suitable for subsequent experiments. As the signals generated in different motion states were different and the corresponding application occasions were also different, the signals in different motion states were recorded, such as walking without swinging arms, walking with swinging arms, running, jumping, and swinging arms only (as illustrated in the accompanying Supplementary Video S1). The voltage and current signals of the W–TENG (Figure 4c,d) and C–ENG (Figure 4e,f) were measured respectively. It can be seen from Figure 4c–f that both voltage and current show the maximum amplitude in the jumping state. The frequency of outputs measured in the running state are obviously higher than that in other states, but the amplitude of outputs decreased to a certain extent. This is because the triboelectric area of the sole with the ground was reduced in the running state. Through a comparison, it can be seen that the detection ability of the C–ENG for the five states is significantly better than the W–TENG. This is because the output performance of the device was greatly improved by coupling the electrostatic induction mechanism. The experiment proved that the C–ENG showed higher resolution for motion states compared with the conventional triboelectric nanogenerator and can precisely and constantly monitor and distinguish various motion states, including stepping, walking, running, and jumping.

### 2.3. The Applications

**Figure 5:**
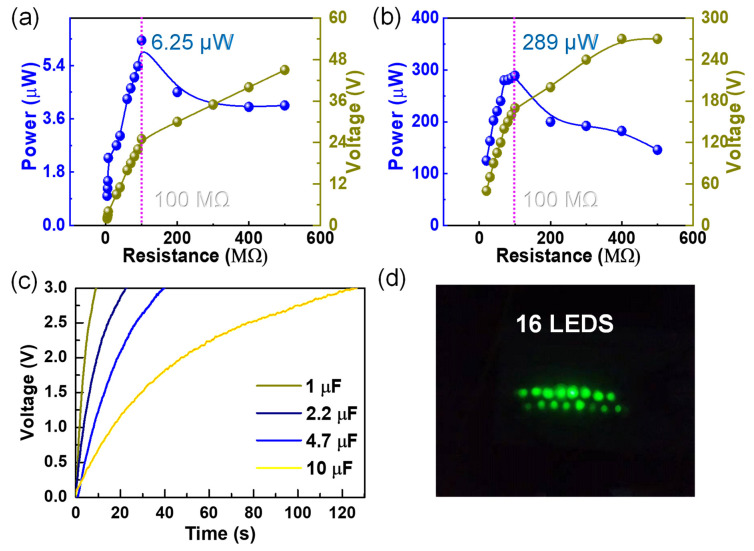
The power and charging characteristics of C–ENG: (**a**) the output power of the W–TENG; (**b**) the output power of the C–ENG; (**c**) the charge curves of the C–ENG for different capacitors; and (**d**) the experiments on lighting LED lights by the C–ENG.

According to the previous results, it is evident that the ouput voltages and currents of the C–ENG were greatly improved compared with those of the W–TENG. The enhancement in peak output power was further studied in walking condition. As illustrated in Figure 5a,b, the peak output power of the W–TENG and C–ENG were measured respectively, and the maximum values and corresponding impedance were marked. A sustainable and enhanced peak power of approximately 289 μW was produced for the C–ENG when the external impedance was 100 MΩ, which gave over a 46-fold enhancement to the conventional single triboelectric conversion mechanism (6.25 μW for W–TENG). Subsequently, the charging rates for different capacitors (1.0 μF, 2.2 μF, 4.7 μF and 10 μF) were studied to verify the excellent output of the C–ENG, as presented in Figure 5c. It is evident that the larger the capacitance, the longer the charging time, which is consistent with the theoretical basis. Furthermore, 16 LEDs were lit in a series to verify the excellent electrical output of the C–ENG, as illustrated in Figure 5d and Supplementary Video S2.

**Figure 6:**
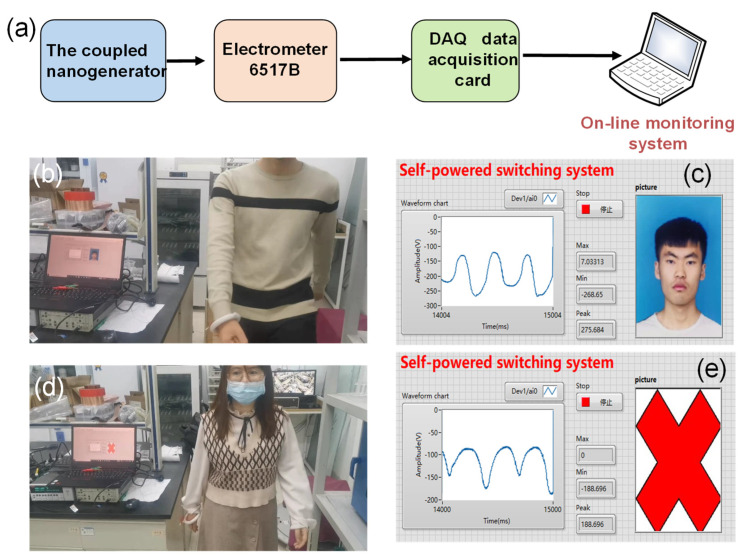
Self-powered switching system: (**a**) the block diagram of the self-powered switching system; (**b**,**c**) the demonstration results of passable personnel; and (**d**,**e**) the demonstration results of impassable personnel.

To demonstrate that the C–ENG can serve as a sensing device for actual scenarios, a self-powered switching system was invented. The system was constructed of the coupled nanogenerator, an electrometer 6517B, a DAQ data acquisition card, and an online monitoring system, which is expressed in Figure 6a. To demonstrate this function, the C–ENG was worn on the wrist to record the voltage waveform output by different people swinging their arms on foot, while simultaneously measuring the maximum and minimum voltage values and calculating the corresponding peak to peak voltage values. The measurement process is illustrated in Figure 6b–e. When the system recognized that the gait information of a person was passable, the information was retrieved from the library and the access control was opened to allow the person to enter, as illustrated in Figure 6b,c, and Supplementary Video S3. In contrast, if the system recognized that the gait information of the personnel was not in the passable range, it called up the blocking command, so as to prevent the invasion of foreign personnel, as illustrated in Figure 6d,e, and Supplementary Video S4. It is worth noting that this part was only a general functional demonstration and the specific application of C–ENG in switching systems needs to be further improved.

**Figure 7:**
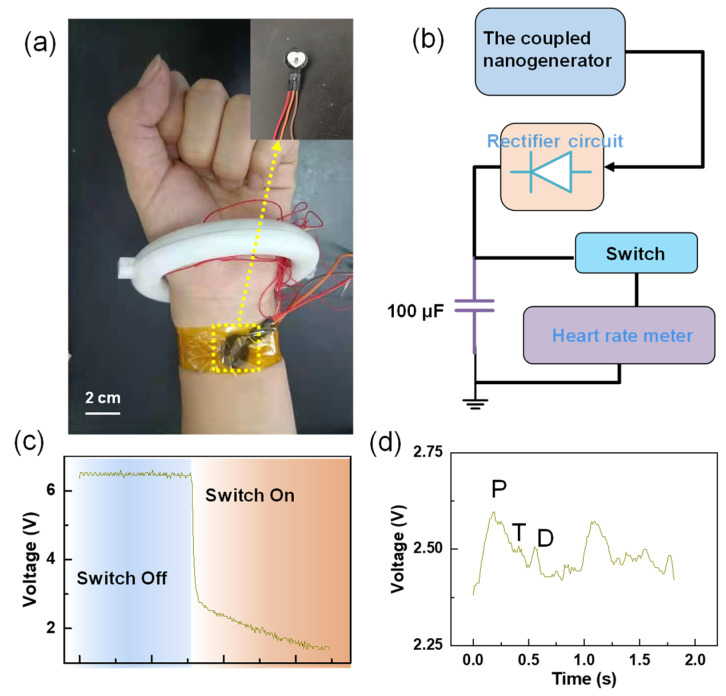
Self-powered heart rate testing system: (**a**) the electronic photo of system; (**b**) the block diagram of system composition; (**c**) the voltage changed at both ends of the capacitor before and after fast closing. After the switch was closed, the electric quantity in the capacitor began to power the heart rate sensor, and the voltage dropped sharply); and (**d**) pulse waveform collected.

In order to verify the application prospect of the C–ENG in the field of self-powered wearable electronics, a self-powered heart rate testing system was built, as illustrated in Figure 7a,b, and Supplementary Video S5. Electric energy was generated by the walking state excitation device, and the generated electrical signal was stored in a 100 μF capacitor after rectification. It transpired that after about 10 min, the capacitor voltage reached 6 V, which is illustrated in Figure 7c. Afterwards, the heart rate test device was connected and the capacitor charging switch was closed. The voltage of the capacitor dropped sharply, as illustrated in Figure 7c. By observing the collected waveform, the single heartbeat can be clearly observed, including the diastolic (D) wave, tidal (T) wave, and percussion (P) wave. This proves that the C–ENG powered the pulse sensor successfully, and the normal working time of the device was two seconds, as illustrated in Figure 7d.

## 3. Conclusions

In summary, a biomechanical energy harvesting device combining triboelectric and electrostatic mechanisms has been demonstrated. Aiming at the low output of energy acquisition devices based on a single conversion mechanism, a scheme of coupling electrostatic energy with triboelectric energy was proposed. The circuit-equivalent model of the coupled energy nanogenerator was constructed and the principle of output lifting was analyzed. Afterwards, the output characteristics of the coupled energy nanogenerator were demonstrated systematically through simulation and experiment. The results show that the output performance of the device was greatly improved by coupling the electrostatic induction mechanism. The best output performance was obtained when the electrode width and spacing were approximately the same as the PTFE ball diameter. The open circuit voltage increased by approximately 12.98 times, while the short circuit current increased by 12.79 times, when the human jumped, when the diameter of the PTFE ball was 6 mm. It is worth emphasizing that when people were walking, a sustainable and enhanced peak power of about 289 μW was produced when the external impedance was 100 MΩ, which gave over a 46-fold enhancement to the conventional single triboelectric conversion mechanism. Moreover, it showed higher resolution of motion states compared with the conventional triboelectric nanogenerator, and can precisely and constantly monitor and distinguish various motion states, including stepping, walking, running, and jumping. Furthermore, the charging rates and power supply features were studied to verify its excellent output, and the result show that it can charge a capacitor of 10 μF to 3 V within 2 min and light up 16 LEDs. After that, a self-powered access control system based on gait recognition was successfully demonstrated. This work provides a more convenient choice for human motion status monitoring, and provides diversified perspectives for personnel identification systems, simultaneously.

## Data Availability

Request the corresponding author of this article.

## Supplementary Materials

The following are available online at www.mdpi.com/article/10.3390/nano12060933/s1: Video S1: the demonstration of gait recognition by the C–ENG; Video S2: the demonstration of lighting 16 LEDs by the C–ENG; Video S3: the demonstration of accessible personnel in access control systems; Video S4: the demonstration of inaccessible personnel in access control systems; and Video S5: the power supply process in a heart rate testing system.

## References

[1] Wei Z., Lin S., Li Q., Song C., Wang F., Tao X.M. (2014). Fiber-based wearable electronics: A review of materials, fabrication, devices, and applications. Adv. Mater..

[2] Han Y.Z., Yi F., Jiang C., Dai K.R., Xu Y.C., Wang X.F., You Z. (2019). Self-powered gait pattern-based identity recognition by a soft and stretchable triboelectric band. Nano Energy.

[3] Zhang Q., Jin T., Cai J.G., Xu L., He T.Y.Y., Wang T.H., Tian Y.Z., Li L., Peng Y., Lee C.K. (2021). Wearable triboelectric sensors enabled gait analysis and waist motion capture for iot-based smart healthcare applications. Adv. Sci..

[4] Liu S., Yuan F., Sang M., Zhou J.Y., Zhang J.S., Wang S., Li J.S., Xuan S.H., Gong X.L. (2021). Functional sponge-based triboelectric nanogenerators with energy harvesting, oil–water separating and multi-mode sensing performance. J. Mater. Chem. A.

[5] Lin Z.M., Wu Z.Y., Zhang B.B., Wang Y.C., Guo H.Y., Liu G.L., Chen C.Y., Chen Y.L., Yang J., Wang Z.L. (2018). A Triboelectric nanogenerator-based smart insole for multifunctional gait monitoring. Adv. Mater. Technol..

[6] Wang H., Cheng J., Wang Z., Ji L., Wang Z.L. (2020). Triboelectric nanogenerators for human-health care. Sci. Bull..

[7] Maiti S., Karan S.K., Kim J.K., Khatua B.H. (2019). Nature driven bio-piezoelectric/triboelectric nanogenerator as next-generation green energy harvester for smart and pollution free society. Adv. Energy Mater..

[8] Lim J., Choi D.S., Lee G.Y., Lee H.J., Sasikala S.P., Lee K.E., Kang S.H., Kim S.O. (2017). Omnidirectional deformable energy textile for human joint movement compatible energy storage. ACS Appl. Mater. Interfaces.

[9] Wu H.Z., Tatarenko A., Bichurin M.I., Wang Y.J. (2021). A multiferroic module for biomechanical energy harvesting. Nano Energy.

[10] Gao L.X., Chen X., Lu S., Zhou H., Xie W.B., Chen J.F., Qi M.K., Yu H., Mu X.J., Wang Z.L. (2019). Enhancing the output performance of triboelectric nanogenerator via grating-electrode enabled surface plasmon excitation. Adv. Energy Mater..

[11] Tcho I.W., Kim W.G., Choi Y.K. (2020). A self-powered character recognition device based on a triboelectric nanogenerator. Nano Energy.

[12] Zhen W., Shen Q., Sun X. (2017). Nanogenerators for self-powered gas sensing. Nano-Micro Lett..

[13] Fu H.L., Mei X.T., Yurchenko D., Zhou S.X., Theodossiades S., Nakano K., Yeatamn E.M. (2021). Rotational energy harvesting for self-powered sensing. Joule.

[14] Chen J., Han K., Luo J.J., Xu L., Tang W., Wang Z.L. (2020). Soft robots with self-powered configurational sensing. Nano Energy.

[15] Zhang B.S., Zhang S., Li W.B., Gao Q., Zhao D., Wang Z.L., Cheng T.H. (2021). Self-powered sensing for smart agriculture by electromagnetic–triboelectric hybrid generator. ACS Nano.

[16] Ma M.Y., Zhang Z., Liao Q.L., Yi F., Han L.H., Zhang G.J., Liu S., Liao X.Q., Zhang Y. (2017). Self-powered artificial electronic skin for high-resolution pressure sensing. Nano Energy.

[17] Askari H., Hashemi E., Khajepour A., Khamesee M.B., Wang Z.L. (2018). Towards self-powered sensing using nanogenerators for automotive systems. Nano Energy.

[18] Xie W.B., Gao L.X., Wu L.K., Chen X., Wang F.Y., Tong D.Q., Zhang J., Lan J.Y., He X.B., Mu X.J. (2021). A non-resonant hybridized electromagnetic-triboelectric nanogenerator for irregular and ultralow frequency blue energy harvesting. Research.

[19] Islam E., Abdullah A.M., Chowdhury A.R., Tasnim F., Uddin M.J. (2020). Electromagnetic-triboelectric-hybrid energy tile for biomechanical green energy harvesting. Nano Energy.

[20] Zou Y.J., Raveendran V., Chen J. (2020). Wearable triboelectric nanogenerators for biomechanical energy harvesting. Nano Energy.

[21] Cheng Y., Gao Y.Y., Zhao S.L., Zhang S.L., Zhou Y.H., Deng W.L., Li Z.W., Jiang G., Jin L., Tian G. (2020). A linear-to-rotary hybrid nanogenerator for high-performance wearable biomechanical energy harvesting. Nano Energy.

[22] Shen J.L., Li Z.L., Yu J.Y., Ding B. (2017). Humidity-resisting triboelectric nanogenerator for high performance biomechanical energy harvesting. Nano Energy.

[23] Zhou Z.H., Weng L., Tat T., Libanori A., Lin Z.M., Ge L.J., Yang J., Chen J. (2020). Smart insole for robust wearable biomechanical energy harvesting in harsh environments. ACS Nano.

[24] Yang Z.B., Zhou S.X., Zu J., Inman D. (2018). High-performance piezoelectric energy harvesters and their applications. Joule.

[25] Zhang S.L., Bick M., Xiao X., Chen G.R., Nashalian A., Chen J. (2021). Leveraging triboelectric nanogenerators for bioengineering. Matter.

[26] Gao L.X., Lu S., Xie W.B., Chen X., Wu L.K., Wang T.T., Wang A.B., Yue C.Q., Tong D.Q., Lei W.Q. (2020). A self-powered and self-functional tracking system based on triboelectric-electromagnetic hybridized blue energy harvesting module. Nano Energy.

[27] Ezzitouni S., Fernández-Yáez P., Sánchez L., Armas O. (2020). Global energy balance in a diesel engine with a thermoelectric generator. Appl. Energy.

[28] Dragunov V.P., Ostertak D.I., Pelmenev K.G., Sinitskiy R.E., Dragunova E.V. (2020). Electrostatic vibrational energy converter with two variable capacitors. Sens. Actuators A Phys..

[29] Feser J.P., Ravichandran J. (2018). More power to pyroelectrics. Nat. Mater..

[30] Garcia C., Trendafilova I. (2019). Real-time diagnosis of small energy impacts using a triboelectric nanosensor. Sens. Actuators A Phys..

[31] Garcia C., Trendafilova I., de Villoria R.G., del Rio J.S. (2018). Self-powered pressure sensor based on the triboelectric effect and its analysis using dynamic mechanical analysis. Nano Energy.

[32] Zou H., Zhang Y., Guo L., Wang P., He X., Dai G., Zheng H., Chen C., Wang A.C., Xu C. (2019). Quantifying the triboelectric series. Nat. Commun..

[33] Niu S., Zhou Y.S., Wang S., Liu Y., Lin L., Bando Y., Wang Z.L. (2014). Simulation method for optimizing the performance of an integrated triboelectric nanogenerator energy harvesting system. Nano Energy.

